# A bivalent recombinant vaccine: a promising strategy against both SARS-CoV-2 variants and wild type of the virus

**DOI:** 10.1038/s41392-021-00691-4

**Published:** 2021-07-17

**Authors:** Junzhi Wang

**Affiliations:** Key Laboratory of the Ministry of Health for Research on Quality and Standardization of Biotech Products, National Institutes for Food and Drug Control, Beijing, China

**Keywords:** Drug discovery, Molecular medicine

A recent study published in Medcomm by He et al.^1^ introduced protein subunit vaccines with wild-type and mutant S1 subunit (S1-WT and S1-Mut) of severe acute respiratory syndrome coronavirus 2 (SARS-CoV-2) to evaluate their immunoprotective efficacy. The team further analyzed the neutralization activity of bivalent vaccine composed of S1-WT and S1-Mut for SARS-CoV-2 and mutant strains to explore a universal protective vaccine.

The coronavirus disease 2019 (COVID-19) is still a life-threatening global problem since the SARS-CoV-2 pandemic. To date, SARS-CoV-2 transmission is still uncontrolled in many parts of the world. As of May 4, 2021, SARS-CoV-2 has caused more than 153 million COVID-19 patients and 3.2 million deaths as well as heavy economic burden and overburdened health systems. Comfortingly, the development of recombinant neutralization antibodies and kinds of vaccines contributes to the control of the COVID-19 pandemic. However, a growing number of SARS-CoV-2 variants with enhanced infectivity/transmissibility and increased ability to circumvent drug and immune control have been reported, which brings about new challenges to control the pandemic.^2^

**Fig. 1:**
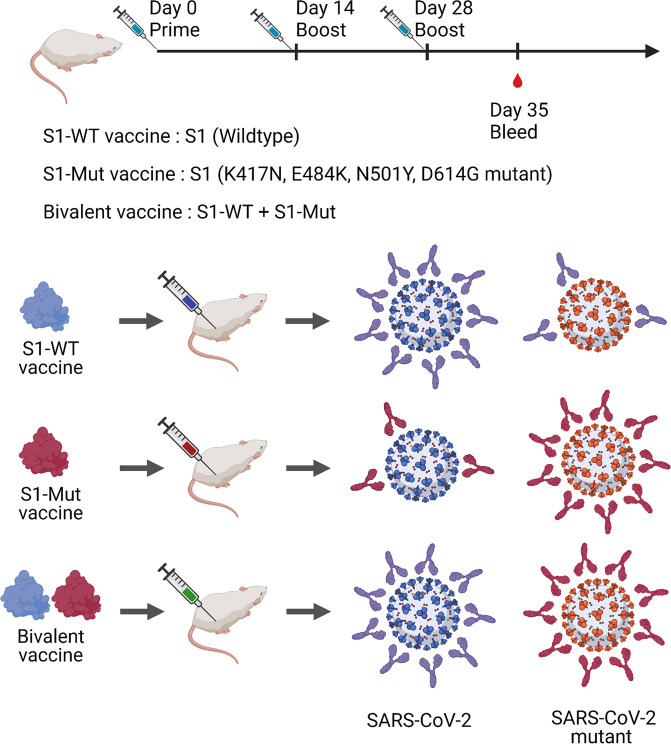
The diagram of monovalent recombinant S1-WT and S1-Mut protein vaccine and the combined bivalent vaccine against SARS-CoV-2 and mutant strains. The ICR-hACE2 mice were immunized with recombinant S1-WT/S1-Mut protein vaccine and bivalent vaccine by intramuscular injection on days 0, 14, and 28, separately. The S1-WT vaccine induced higher neutralizing antibodies against wild-type of SARS-CoV-2 than that of variants while S1-Mut vaccine stimulated higher neutralizing antibodies against SARS-CoV-2 variants. The bivalent recombinant vaccine composed of S1-WT and S1-Mut could induce the neutralizing antibodies against both SARS-CoV-2 variants and wild-type of the virus, indicating a potential and effective approach for the development of universal coronavirus vaccine

A universal coronavirus vaccine against both SARS-CoV-2 and mutant strains has been called for based on the emerging variants because of the decreased protective role of existing vaccines against mutant strains. As reported, the Novavax NVX-CoV2373 subunit vaccine showed a reduced efficacy from 89.3% to 49.4% in clinical studies in South Africa.^3^ Moreover, the protective efficacy of ChAdOx1 chimpanzee adenoviral vectored vaccine (AZD1222) declined more significantly, and the vaccine efficacy against B.1.351 was only 10.4%.^4^ To determine the cross-protection of recombinant S1-WT and S1-Mut protein vaccine against wild-type and mutant SARS-CoV-2, He et al.^1^ chose S1-WT and S1-Mut including K417N, E484K, N501Y and D614G that appear in the current main SARS-CoV-2 mutant strains (B.1.1.7, B.1.351, and P.1) to formulate the recombinant protein vaccine, and they discovered that the geometric mean titers (GMT) of RBD-WT specific antibodies in serum from S1-WT immunized mice was 8.65×10^6^ while the GMT of RBD-Mut antibodies was 1.08 × 10^6^. Besides, the GMT of S1-Mut antibodies in serum from S1-Mut immunized mice was much higher than that of S1-WT immunized mice (1.08 × 10^6^ vs 2.7 × 10^5^), indicating that S1-WT protein stimulates stronger S1-WT and RBD-WT specific antibody responses, and S1-Mut protein could induce mutant S1 and RBD antibody responses. In addition, the neutralization antibodies induced by recombinant S1-WT or S1-Mut protein displayed strong blockade on the binding between ACE2 and RBD-WT or RBD-Mut, respectively. Moreover, the sera from S1-WT immunized mice showed protective effects against wild-type, D614G, B.1.1.7 pseudoviruses but significantly decreased protection against B.1.351 and P.1. In contrast, sera from S1-Mut immunized mice showed higher protection against B.1.351 and P.1 than wild-type and B.1.1.7 pseudoviruses, suggesting the weakened cross-protection of S1-WT and S1-Mut against wild-type and mutant strains (Fig. 1). These results are consistent with the previous reports that the mutations in spike protein, especially in RBD, could induce immune escape, increase the binding of the virus to ACE2, and decrease the efficacy of vaccines based on non-mutant SARS-CoV-2, which indicates that the development of a universal vaccine is extremely urgent.^4,5^

Next, to explore a vaccine exerting strong protection against both wild-type and mutant strains of SARS-CoV-2, the authors combined the recombinant S1-WT and S1-Mut proteins to form a bivalent vaccine. Further, they found that the bivalent vaccine demonstrated cross-protection against wild-type and mutant SARS-CoV-2, which indicates that the bivalent vaccine might be a candidate as a universal vaccine against SARS-CoV-2 and mutant strains (Fig. 1). And the safety of the bivalent vaccine was good in mice. Despite the lack of data from other models, these results are still encouraging and might provide a promising strategy to explore the potential clinical use of the bivalent recombinant vaccine to prevent the COVID-19 pandemic in the future.

Taken together, the study by He and colleagues highlights the immunoprotection of wild-type and mutant S1 proteins in hACE2 mice, and points that the monovalent recombinant protein vaccines are not universal for both SARS-CoV-2 and mutant strains. Moreover, the authors suggest the bivalent vaccine as a potential strategy, which shows excellent neutralization against wild-type and mutant pseudoviruses. Critically, the authors used various pseudoviruses rather than viruses to determine the neutralization property of these recombinant proteins, which may not precisely represent the data profile in live viruses. Therefore, further studies are warranted to characterize the protective role of the bivalent vaccine in nonhuman animals against SARS-CoV-2 and mutant strains. The authors were devoted to exploring cross-protective bivalent fusion vaccines that consist of wild-type and mutant spike protein fragments, which might be another approach to prevent SARS-CoV-2 and mutant strains. Nevertheless, it is very appealing to concentrate on this promising strategy to explore universal coronavirus vaccine against both SARS-CoV-2 and mutant strains. The further development and application of the universal coronavirus vaccine may offer an effective, promising strategy for the control and prevention of the fast growing worldwide pandemic.
